# Everolimus‐containing therapy vs conventional therapy in the treatment of refractory breast cancer patients with *PI3K/AKT/mTOR* mutations: A retrospective study

**DOI:** 10.1002/cam4.2460

**Published:** 2019-08-06

**Authors:** Zhanhong Chen, Yabing Zheng, Wenming Cao, Yuzi Zhang, Zhengyi Zhao, Guoqiang Wang, Jing Zhao, Shangli Cai, Xiying Shao, Jian Huang, Weiwu Ye, Yuan Huang, Wei Li, Xiang Huang, Hao Wu, Xiaojia Wang, Yongmei Yin

**Affiliations:** ^1^ Department of Breast Medical Oncology Zhejiang Cancer Hospital Hangzhou China; ^2^ The Medical Department 3D Medicines Inc Shanghai China; ^3^ Department of Oncology The First Affiliated Hospital of Nanjing Medical University Nanjing China

**Keywords:** breast cancer, everolimus, mutation, PI3K/AKT/mTOR, prognosis

## Abstract

**Background:**

Previous case reports have shown the promising antitumor activity of everolimus in solid tumors containing molecular aberrations in *PI3K/ATK/mTOR* pathway, however, whether it is effective in patients with breast cancer remains unknown. Therefore, we conducted this retrospective cohort study to compare the efficacy of molecularly matched targeted therapy with everolimus to conventional therapy in refractory breast cancer patients harboring *PI3K/ATK/mTOR* pathway activating mutations.

**Methods:**

Refractory metastatic breast cancer patients who have received molecular screening using next‐generation sequencing (NGS) between September 8, 2015 and October 30, 2017 in two sites were screened for this study. The primary outcome was progression‐free survival (PFS). The secondary outcomes were overall response rate (ORR), disease control rate (DCR), and safety profile.

**Results:**

A total of 78 patients were screened for analysis, amongst all, 52 (66.7%) had at least one gene mutation in *PI3K/AKT/mTOR* pathway. The most common mutation fell in *PIK3CA* (76.9%, 40/52) with a mutational prevalence of 51.3%. Of the 32 patients who were eligible for efficacy analysis, patients in the everolimus group (n = 19) exhibited shorter PFS than those in the conventional group (n = 13) (median, 1.9 vs 6.1 months; HR, 3.6; 95% CI, 1.48‐8.81; *P* = .0005). ORR was 15.4% (2/13) in the everolimus group and 23.1% (3/13) in the conventional group (*P* = 1.000), and DCR was 30.8% (4/13) and 100% (13/13) for each group, respectively (*P* = .000). The incidence of grade 3‐5 adverse events was relatively higher in the conventional group (38.5%, 5/13) than that in the everolimus group (26.3%, 5/19).

**Conclusions:**

Our findings suggested that everolimus might not be effective for cancer patients harboring mutations in *PI3K/ATK/mTOR* pathway and physicians should be cautious about its off‐label use in clinical practice.

## Graphical abstract

About 66.7% of patients with refractory breast cancer had at least one gene mutation in PI3K/AKT/mTOR pathway. Off‐label use of everolimus might not provide sufficient benefit for refractory breast cancer patients harboring PI3K/AKT/mTOR pathway mutations.

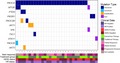

## 1 INTRODUCTION

Most of the patients with metastatic breast cancer (MBC) eventually exhausts standard therapeutic options, fortunately, emerging evidences showing the benefit from matched targeted regimens have provided promising strategies to solve this problem.^1^ With the approval of next‐generation sequencing (NGS) tests by Food and Drug Administration (FDA),^2^ molecular screening using NGS to identify genomic alterations that can be targeted in patients with refractory MBC is becoming increasingly common in clinical practice.^3^

In general, there is a considerable proportion of cancer patients (34.9%‐51.6%) carrying at least one genomic alteration that could be targeted by approved drugs.^4^, ^5^, ^6^, ^7^ The efficacy of personalized treatment given on the basis of genomic molecular profiling has been assessed in several studies, especially for patients with refractory tumors. Previous studies reported that nearly one‐third of refractory cancer patients experienced an improvement in progression‐free survival (PFS) on molecularly matched therapy.^5^, ^6^, ^7^, ^8^, ^9^, ^10^ However, clinical trials that were designed to evaluate the rationale therapy based on tumor molecular profiling, for example, SHIVA and WINTHER study, failed to prove the benefit of matched cancer treatment.^4^, ^11^ In addition, limited antitumor activity was observed in arms Q and I from the phase II trial NCI‐MATCH, which evaluated the efficacy of T‐DM1 in human epidermal growth factor‐2 (HER2) amplified tumors and taselisib in PIK3CA mutated tumors, respectively.^12^, ^13^ Similarly, controversial results were also presented in MBC specific studies. One pilot study reported a 30% increase in the PFS ratio for 44% of the MBC patients.^14^ However, the prospective single‐arm trial (SAFIR01/UNICANCER) showed the disappointing objective response rate (ORR) of only 9% with genotype‐directed therapy.^15^ Moreover, study from Pezo and colleagues also suggested that matched therapy failed to provide benefit for MBC patients compared with unmatched therapy.^16^

Genes involved in *PI3K/ATK/mTOR* pathway are frequently altered across a variety of tumors and are considered as actionable targets.^4^, ^6^, ^7^, ^8^, ^9^, ^10^ Activation in *PI3K/ATK/mTOR* pathway is also considered to be the resistant mechanism of endocrine therapy and trastuzumab therapy in breast cancer.^17^, ^18^, ^19^, ^20^ As such, combination therapies of everolimus, an *mTORC1* inhibitor, with endocrine therapy such as exemestane, fulvestrant, and tamoxifen are now standard treatment for postmenopausal females with hormone receptor positive, HER2 negative (HR+ HER2‐) MBC.^21^ A series of studies and case reports indicated that tumors with alterations in *PI3K/ATK/mTOR* pathway exhibit sensitivity to everolimus in patients with triple negative breast cancer, HER2 positive breast cancer, and gastric cancer, etc.^22^, ^23^, ^24^, ^25^, ^26^, ^27^ Therefore, everolimus is often used in off‐label to target *PI3K/ATK/mTOR* pathway in patients with refractory cancer. However, results from the secondary biomarker analysis of BOLERO‐2 study suggested that everolimus benefit was independent of PIK3CA gene status in HR+ HER2‐ breast cancer,^28^, ^29^ in line with the results from the phase II SHIVA trial, showing that patients harboring *PI3K/ATK/mTOR* pathway alterations did not benefit from matched everolimus treatment compared with treatment at physician's choice (HR 0.79, 95% CI 0.51‐1.24, *P* = .30).^4^ Taken together, the benefit of everolimus in refractory breast cancer patients harboring activation alterations in *PI3K/ATK/mTOR* pathway remains unclear.

Therefore, we conducted this retrospective study to assess the efficacy of molecularly matched off‐label use of everolimus compared with conventional therapy in refractory breast cancer patients with active mutations in *PI3K/ATK/mTOR* pathway.

## 2 METHODS

### 2.1 Patients and treatment

**Figure 1:**
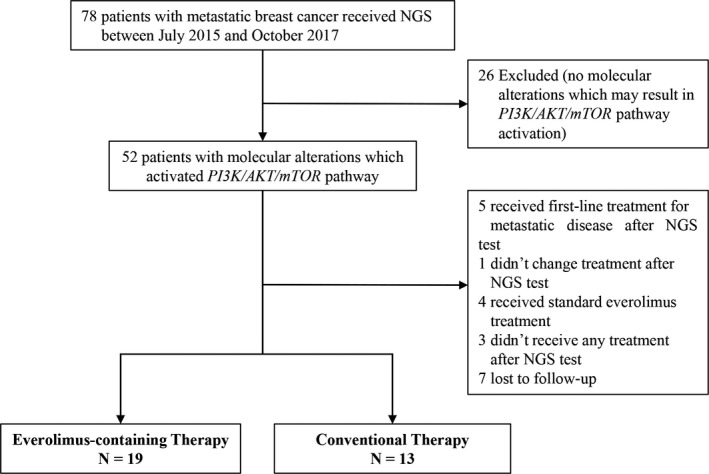
Patient selection diagram

Patients with refractory MBC who received next‐generation sequencing (NGS) with the aim of guiding treatment after confirmed disease progression between September 8, 2015 and October 30, 2017 were screened at two sites in China (Zhejiang Province Cancer Hospital [ZPCH] and the First Affiliated Hospital of Nanjing Medical University [FAHNMU]) (Figure 1). To be included, patients needed to have at least one established gene mutation resulted in *PI3K/AKT/mTOR* pathway activation and received everolimus containing therapy or conventional therapy after NGS testing. The 19 genes involved in *PI3K/AKT/mTOR* pathway are shown in Table S1. Given that standard combination treatment of everolimus with exemestane/fulvestrant/tamoxifen have been proved to be effective in HR+ HER2‐ breast cancer patients from randomized trials, patients who received off‐label use of everolimus were eligible for this analysis. Everolimus was given in accordance with the product information. Conventional therapy was defined as physicians’ choice of chemotherapy. Treatment choices were made by the physicians based on patient's physical condition, prior treatment efficacy, molecular profiling, toxicity, healthcare coverage, and patient's decision. Given that pertuzumab, T‐DM1, and CDK4/6 inhibitors were not approved for clinical use in China during this period, these drugs were not available to most of the Chinese patients. In addition, patients who harbored HER2 amplification and have progressed after anti‐HER2 therapy should be treated with the combination of anti‐HER2 therapy in this analysis. Detailed inclusion and exclusion criteria were shown in Table S2. Written informed consent was provided by all patients under approval of the Institutional Review Board of ZPCH and FAHNMU.

This was a retrospective analysis of prospectively collected data. Based on the predesigned case report form (CRF), clinical data from the electronic medical record database of each patients were extracted and verified independently by two oncologist physicians.

### 2.2 Genomic analysis

Formalin fixed paraffin‐embedded (FFPE) tumor specimens or fresh tumor tissues were sent for NGS testing in 3DMed Clinical Laboratory Inc, a College of American Pathologists (CAP) certified and Clinical Laboratory Improvement Amendments (CLIA) certified laboratory of 3D Medicines Inc. Tissue DNA was processed and 381 cancer‐associated genes (including the 19 genes involved in *PI3K/AKT/mTOR* pathway) were sequenced in the NGS platform Illumina Nextseq 500 to >500X coverage as previously described.^30^ Tumor samples from primary surgery or blood samples could also be used as a substitute when a metastatic site biopsy or resection was not accessible or the biopsy sample was not qualified for NGS testing. Circulating tumor DNA (ctDNA) was processed and 150 cancer‐associated genes (include the 19 genes involved in *PI3K/AKT/mTOR* pathway) were sequenced in the NGS platform Illumina Nextseq 500 to >1000X coverage as previously reported.^31^

### 2.3 Response assessment

The objective tumor response was assessed every 8 weeks routinely in clinical practice. If any symptoms or signs suggesting a progressive disease was observed at any time, one extra assessment could be performed. Tumor size measurement using radiologic imaging was conducted by radiologists from ZPCH and FAHNMU. Assessment of objective response was confirmed by physicians from these two centers per RECIST version 1.1 and the date of disease progression was documented. Based on the predesigned CRF, physicians collected the objective response assessment and the disease progression date retrospectively from the medical records.

### 2.4 Outcomes

The primary outcome was PFS, defined as the time from the initiation of everolimus or conventional therapy to the date of progression according to RECIST 1.1, or death from any cause or last contact (censored). Secondary outcomes were ORR and safety. Adverse events and laboratory abnormalities were graded *per* the National Cancer Institute Common Terminology Criteria for Adverse Events (NCI CTCAE, version 4.0).

### 2.5 Statistical analysis

Continuous or ordinal variables were described as mean ± SD using T test when normally distributed and otherwise described as median ± SD using the Mann‐Whitney U test. The categorical variables were compared by Chi‐square or Fisher's exact test. The Kaplan‐Meier method was used to estimate PFS. Differences in PFS was assessed with a log‐rank test. Hazard ratios (HR) and associated 95% confidential intervals (95% CIs) were determined by Cox's regression. The missing data were not analyzed. All reported P values were two‐sided, and *P* < .05 was considered statistically significant, unless otherwise specified.

The multivariable Cox proportional hazards regression model was used to evaluate the prognostic role of everolimus therapy after adjusting for other risk factors which might be related to PFS. Variables that achieved *P* ≤ .05 in the univariable analysis or might have an important effect on prognosis were entered into multivariable models.

All analysis were performed using GraphPad Prism (version 7.01; GraphPad Software), SPSS statistical software (version 20.0; SPSS, IBM Corporation) or R (version 4.3.1; R Development Core Team).

## 3 RESULTS

### 3.1 Patient characteristics

Of all 78 patients, 32 patients were eligible for this analysis. Amongst all, 19 (59.4%) patients received everolimus‐containing therapy and 13 patients (40.6%) were treated with conventional therapy. Treatment strategy received by each individual in the everolimus group is shown in Table S3. Baseline characteristics were not well‐balanced between two groups. Proportions of both HER2 + and HR‐ population were higher in the everolimus group than the conventional group. Moreover, there are relatively more patients with liver metastasis in the conventional group (Table 1). It is important to note that patients in this study were refractory and hard‐to‐treat, as all of the patients had progressed after previous chemotherapy for metastatic disease and a large proportion of them had visceral metastasis.

**Table 1:** Characteristics of the patients at baseline

Characteristic	Everolimus therapy (N=19)	Conventional therapy (N = 13)	*P* value
Age, Mean ± SD, years (range)	47 ± 12 (22‐68)	54 ± 9 (40‐70)	.120
Menopausal status			
Premenopausal	9 (47.4%)	4 (30.8%)	.702
Postmenopausal	9 (47.4%)	9 (69.2%)	
Unknown	1 (5.2%)	0 (0.0%)	
ECOG performance status			
0	9 (47.4%)	2 (15.4%)	.184
1	7 (36.8%)	7 (53.8%)	
2	2 (10.6%)	1 (7.7%)	
3‐4	1 (5.2%)	3 (23.1%)	
Tumor histology			
Ductal invasive	15 (78.9%)	12 (92.3%)	.625
Other	4 (21.1%)	1 (7.7%)	
Hormone‐receptor status			
Estrogen‐receptor or progesterone‐receptor positive	8 (42.1%)	9 (69.2%)	.131
Estrogen‐receptor negative and progesterone‐receptor negative	11 (57.9%)	4 (30.8%)	
Human epidermal growth factor receptor 2 status			
Positive	11 (57.9%)	3 (23.1%)	.051
Negative	8 (42.1%)	10 (76.9%)	
Previous systemic treatment for metastatic disease			
Chemotherapy	19 (100%)	13 (100%)	
Endocrine therapy	4 (21.1%)	6 (46.1%)	.244
Anti‐HER2 therapy	11 (57.9%)	3 (23.1%)	.051
Prior lines for metastatic disease			
1	4 (21.1%)	4 (30.8%)	.819
2	7 (36.8%)	4 (30.8%)	
≥3	8 (42.1%)	5 (38.4%)	
Metastatic site			
Breast	2 (10.6%)	2 (15.4%)	.660
Bone	8 (42.1%)	5 (38.5%)	.821
Visceral			
Lung	11 (57.9%)	7 (53.8%)	.821
Brain	1 (5.2%)	1 (7.7%)	1.000
Liver	6 (31.6%)	8 (61.5%)	.093
Specimen type			
Tissue	17	10	.374
ctDNA	2	3	

### 3.2 PI3K/AKT/mTOR pathway mutation status

In overall, 65 patients have received tissue test (including 45 patients with biopsies of metastatic sites and 20 patients with primary tumor block) and 13 patients received ctDNA test. A total of 52 (66.7%) had at least one gene mutation that resulted in *PI3K/AKT/mTOR* pathway activation, and 15 (28.8%) of these 52 patients harbored multiple alterations in this pathway. As expect, the most frequent mutation was *PIK3CA* in the pathway (76.9%, 40/52), with a mutational prevalence of 51.3% (40/78) in the overall population. Of the 32 patients who were eligible for efficacy analysis, 27 patients received tissue test (including 18 patients with biopsies of metastatic sites and nine patients with primary tumor block) and five patients received ctDNA test. A total of 9 (28.1%) patients had two or more mutations in *PI3K/AKT/mTOR* pathway, but none had more than four mutations (Table S4). Of these nine patients, six had estrogen receptor or progesterone receptor expression and seven harbored HER2 gene amplification. All of the HER2‐positive patients have progressed after anti‐HER2 therapy for metastatic disease.

### 3.3 Efficacy analysis

**Figure 2:**
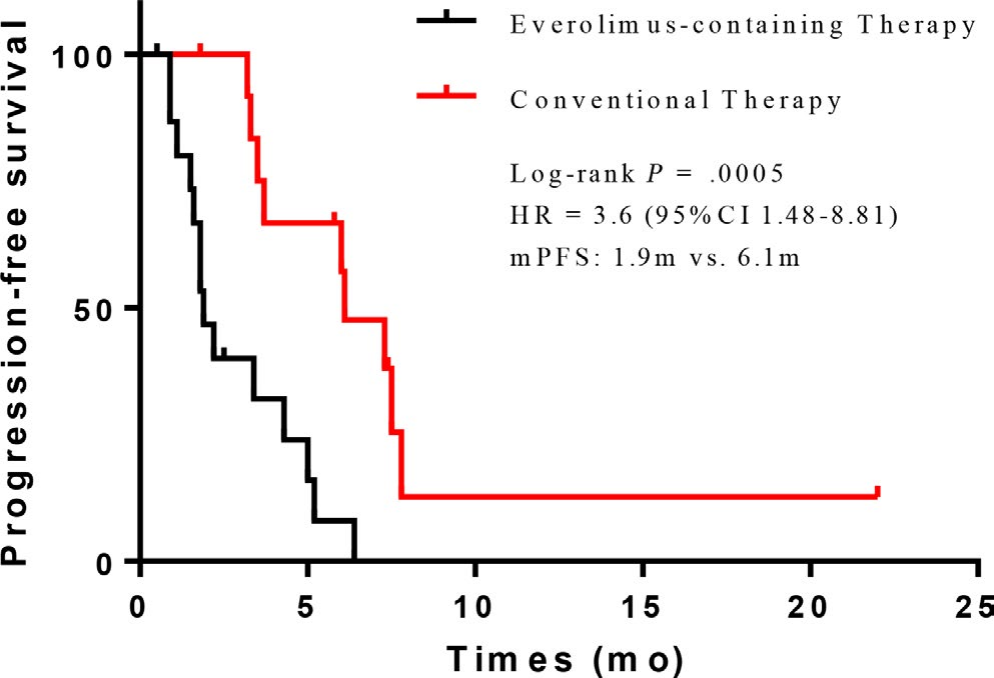
Kaplan‐Meier estimates of progression‐free survival. Kaplan‐Meier survival curves of progression‐free survival comparing everolimus‐containing therapy and conventional therapy

**Figure 3:**
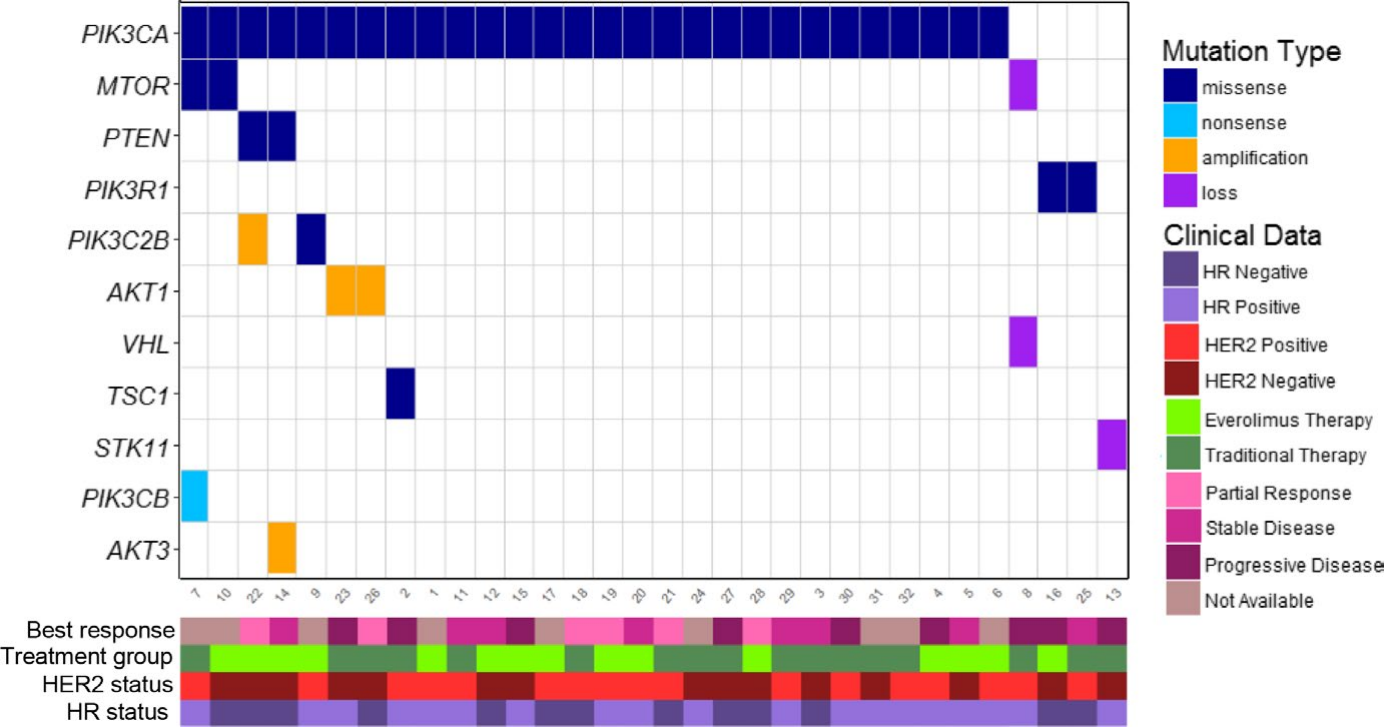
Genomic aberrations in *PI3K/AKT/mTOR* pathway in patients with refractory breast cancer. Data were shown for 32 patients who were included inthis analysis

The median follow‐up period was 11.0 (interquartile range 5.9‐ 21.5) months. Median progression‐free survival was 1.9 months in the everolimus group vs 6.1 months in the conventional group (HR, 3.6; 95% CI 1.48‐8.81; *P* = .0005) (Figure 2). In the multivariable model including age, hormone‐receptor status, HER2 status, and treatment group, treatment group was the only independent predictors for PFS (HR, 2.24; 95% CI 1.34‐3.75; *P* = .002) (Table 2). ORR was 15.4% (2/13) in the everolimus group and 23.1% (3/13) in the conventional group (*P* = 1.000) (Table 3). No complete response was observed. Both of the patients who showed response in the everolimus group were identified to carry a *PIK3CA* mutation (Figure 3). Of the three patients who showed response in the conventional group, two had a *PIK3CA* mutation and one had a *PIK3R1* mutation. DCR was 30.8% (4/13) in the everolimus group and 100% (13/13) in the conventional group (*P* = .000). All of the patients who had a stable disease status harbored a *PIK3CA* mutation.

**Table 2:** Univariable and multivariable analysis of progression‐free survival

Parameter	Univariable analysis			Multivariable analysis		
	HR	95% CI	*P* value	HR	95% CI	*P* value
Age						
<65 vs ≥65	1.50	0.43‐5.17	.523	1.18	0.43‐5.17	.801
Menopausal status						
Premenopausal vs postmenopausal	1.45	0.51‐4.11	.486			
ECOG performance status						
0‐1 vs 2‐4	0.989	0.39‐2.54	.981			
Tumor histology						
Invasive ductal carcinoma vs other	0.569	0.18‐1.76	.326			
Hormone‐receptor status						
Positive vs negative	2.07	0.90‐4.75	.088	0.40	0.14‐1.13	.085
Human epidermal growth factor receptor 2 status						
Positive vs negative	1.22	0.53‐2.77	.640	0.74	0.44‐1.24	.255
Previous systemic treatment for metastatic disease						
Endocrine therapy (yes vs no)	1.38	0.86‐2.20	.186			
Anti‐HER2 therapy (yes vs no)	1.22	0.53‐2.77	.640			
No. of previous lines systemic therapy for metastatic disease						
1 vs ≥2	0.888	0.345‐2.29	.806			
Metastatic site						
Breast (yes vs no)	2.36	0.54‐10.23	.252			
Bone (yes vs no)	1.02	0.44‐2.37	.968			
Visceral						
Lung (yes vs no)	1.21	0.51‐2.90	.663			
Brain (yes vs no)	0.853	0.20‐3.73	.833			
Liver (yes vs no)	1.35	0.58‐3.12	.490			
Treatment group						
Everolimus vs conventional	3.60	1.48‐8.81	.0005	2.24	1.34‐3.75	.002

**Table 3:** Response assessed per RECIST version 1.1

	Everolimus therapy (N = 13)	Conventional therapy (N = 13)
Objective response, n (%; 95% CI)	2 (15.4%; 2.82‐40.93)	3 (23.1%; 6.57‐49.46)
Estimated difference, % (95% CI)	7.7% (−22.47‐37.87)	
*P* value	1.000	
Disease control rate, n (%; 95% CI)	4 (30.8%; 11.26‐57.27)	13 (100.0%)
Estimated difference, % (95% CI)	69.2% (44.10‐94.30)	
*P* value	0.000	
Best overall response, n (%)		
Complete response	0	0
Partial response	2 (15.4%)	3 (21.4%)
Stable disease	2 (15.4%)	10 (78.6%)
Progressive disease	9 (69.2%)	0 (0%)

### 3.4 Adverse events

Grade 3‐5 treatment‐related adverse events were observed in 5 (26.3%) of the 19 patients who received everolimus therapy and 5 (38.5%) of the 13 patients who received conventional therapy (*P* = .699) (Table 4). The most common adverse events were stomatitis and anemia in the everolimus group and neutropenia and anemia in the conventional group. No treatment‐related deaths were observed for either group.

**Table 4:** Adverse events

	Everolimus therapy (N = 19)				Conventional therapy (N = 13)			
	Grade 1‐2	Grade 3	Grade 4	Grade 5	Grade 1‐2	Grade 3	Grade 4	Grade 5
Treatment related								
Any	9 (47.4%)	3 (15.8%)	2 (10.5%)	0	6 (46.2%)	4 (30.8%)	1 (7.7%)	0
Anemia	4 (21.1%)	0	1 (5.3%)	0	4 (30.8%)	0	0	0
Stomatitis	3 (15.8%)	2 (10.5%)	0	0	0	0	0	0
Leukopenia	2 (15.5%)	0	1 (5.3%)	0	1 (7.7%)	3 (23.1%)	0	0
Neutropenia	2 (10.5%)	0	1 (5.3%)	0	3 (23.1%)	1 (7.7%)	0	0
Increased alanine aminotransferase	1 (5.3%)	0	0	0	1 (7.7%)	1 (7.7%)	0	0
Increased aspartate aminortransferase	1 (5.3%)	0	0	0	1 (7.7%)	1 (7.7%)	0	0
Vomiting	1 (5.3%)	1 (5.3%)	0	0	0	0	0	0
Thrombocytopenia	1 (5.3%)	0	0	0	0	0	0	0
Hyperglycemia	1 (5.3%)	0	0	0	0	0	0	0
Pneumonitis	0	1 (5.3%)	0	0	0	0	0	0
Nausea	0	1 (5.3%)	0	0	0	0	0	0
Headache	0	1 (5.3%)	0	0	0	0	0	0
Fatigue	0	0	0	0	0	1 (7.7%)	0	0
Renal function abnormal	0	0	0	0	0	1 (7.7%)	1 (7.7%)	0
Rash	0	0	0	0	0	0	0	0
Hypertriglyceridemia	0	0	0	0	0	0	0	0
Hypercholesterolemia	0	0	0	0	0	0	0	0
Fever	0	0	0	0	0	0	0	0
Myalgia	0	0	0	0	0	0	0	0

## 4 DISCUSSION

Molecular alterations of genes in *PI3K/ATK/mTOR* pathway are considered to be involved in the mechanisms of resistance to a variety of treatments^32^ and everolimus has been frequently used to target alterations in this pathway. However, this observational study suggested that the use of everolimus to target *PI3K/ATK/mTOR* pathway may not improve the clinical outcome of refractory MBC patients compared with conventional therapy.

Some studies evaluating the efficacy of molecularly matched therapy have been reported in MBC patients. Jameson and colleagues’ pilot study reported that 28.6% (8/28) of MBC patients had *PTEN* alteration and 7.1% (2/28) had *PIK3CA* alteration based on protein and transcriptomics profiling results from c‐DNA microarray, IHC, FISH, and RPPA analysis.^14^ In SAFIR01/UNICANCER study, it was shown that 25% of MBC patients harboring alterations in *PIK3CA* exon 10 or exon 21 by comparative genomic hybridization (CGH) and Sanger sequencing.^15^ Mutations in *AKT1* (exon 4) and amplifications in *AKT2* were less common, with a frequency of 4% and 2%, respectively.^15^ However, only 9% of the patients were reported to achieve objective response upon the molecularly matched treatment, including one carrying *AKT2* amplification with *AKT1* and/or mTOR inhibitor treatment.^15^ More recently, Pezo and colleagues identified a mutational frequency of 28% in *PIK3CA* from 440 MBC patients using three sequencing panels that covered only the hotspot mutation regions of 23‐48 cancer‐related genes.^16^ A total of 29 patients were identified to have *PIK3CA* mutation and five of them achieved partial response in PI3K inhibitor clinical trials with an ORR of 16.7%. Unfortunately, matched therapy failed to show clinical benefit vs unmatched therapy group. In our study, 66.7% (52/78) of the patients carried at least one alteration in *PI3K/ATK/mTOR* pathway and the most frequently mutated gene in the pathway was *PIK3CA* with a prevalence of 51.3%, which is higher than the previous report. The difference of mutational rate may be caused by several reasons. First, patients included in our study were heavily treated and resistant to several prior lines of treatments. The high proportion rate of *PIK3CA* mutation may reflect the drug‐resistant status and the refractory features of the population. Second, we used an NGS panel including 19 genes involved in *PI3K/AKT/mTOR* pathway for target screening, which covered all exons of *PIK3CA*, not only selective exons or hotspots, as reported in some of the previous studies. Third, the NGS in our study was conducted with a coverage of 500X for tissue and >1000X for ctDNA, which was sufficient to characterize rare sequence variants and identify mutations within subclones given the small proportion of somatic mutations presented in the tumor cells. Based on our results, 2 of 13 patients achieved partial response (one with *PIK3CA* mutation and another with *STK11* copy number loss), but no significant difference was observed between everolimus group and conventional group.

Controversial results were reported in other studies. For example, IMPACT study, ProfiLER study (with a 69 gene NGS panel and aCGH),^5^ MOSCATO 01 study (with RNA sequencing, aCGH, and whole‐exome sequencing),^9^ and another extensive molecular screening program (with a 426 cancer‐related NGS panel)^6^ have demonstrated improved clinical outcomes of matched therapy. Nevertheless, WINTHER trial (with NGS and mRNA expression sequencing)^11^ and SHIVA trial (with IHC and NGS)^4^ failed to prove favorable benefit with genotype‐directed therapy. More specifically, in the *PI3K/AKT/mTOR* pathway subgroup (accounted for 46.7% of the overall population) analysis of the SHIVA trial, no significant difference in PFS was observed in the everolimus group vs control group (median 2.4 vs. 1.9 months; HR 0.79, 95% CI, 0.51‐1.24, *P* = .30).^4^ Similarly, in the present study, we observed inferior PFS in the everolimus group compared with the conventional therapy group in MBC patients with *PI3K/ATK/mTOR* pathway mutations (median 1.9 vs. 6.1 months; HR 3.6, 95% CI, 1.48‐8.81, *P* = .0005). In fact, as an mTORC1 inhibitor, everolimus may not be effective to target the different levels of activation in *PI3K/ATK/mTOR* pathway. In other words, not all alterations in *PI3K/ATK/mTOR* pathway could result in the activation of *mTORC1* and mediate the benefit from everolimus.^33^

Other preclinical studies have indicated that activation in *PI3K/ATK/mTOR* pathway could result in trastuzumab resistance. A secondary exploratory biomarker analysis of BOLERO‐1 and BOLERO‐3 suggested that HER2 positive MBC patients with hyperactive *PI3K/ATK/mTOR* pathway defined as *PIK3CA* mutations and/or *PTEN* loss and/or *AKT1* mutation could drive greater clinical benefit from everolimus therapy when combined with trastuzumab and chemotherapy.^24^ In the present study, patients in HER2 positive subgroup (44%, 14/32) exhibited shorter PFS than those in conventional group (median, 1.4 vs 7.8 months; *P* = .0064). Considering that two HER2 positive patients were treated with everolimus combined with trastuzumab without chemotherapy, which might result in an inferior efficacy (median PFS is 1.8 and 1.1 months, respectively), the analysis was reconducted with the two patients excluded, which showed a consistently inferior PFS in everolimus group compared with conventional group (median 3.4 vs. 7.8 months, *P* = .0091). In sum, our findings indicated that everolimus might not offer sufficient benefits to restore the resistant to trastuzumab therapy for heavily treated refractory, HER2 positive MBC patients.

The median PFS in everolimus therapy group of this study (1.9 month) was shorter than that of the matched therapy group as previously reported,^4^, ^16^ which may be due to several reasons. First, results from BOLERO‐3 suggested that patients without visceral involvement could drive greater benefit from everolimus treatment. While in our study, 89% of the patients in everolimus group had visceral metastasis and 42.1% have received at least three prior lines of treatment. Second, some patients received everolimus monotherapy or in combination with trastuzumab without a chemotherapy regimen, and thus may exert mild efficacy. Third, all patients included for the efficacy analysis carried alterations in *PI3K/ATK/mTOR* pathway, which were reported to be associated with poor prognosis as indicated by IMPACT study.^7^ Besides, the incidences of pneumonitis and grade 3‐5 hematological adverse events in the everolimus group were relatively lower in the studied cohort as compared to the previous reports,^34^, ^35^ which may due to the heavily treated MBC patients with active alterations in *PI3K/ATK/mTOR* pathway in our study and the fact that everolimus was used outside its indications.

For patients with breast cancer who have progressed after standard therapies and run out of treatment options, molecular screening by NGS test is often suggested in clinic to guide therapy. Consequently, patients and physicians are both willing to try the targeted drugs, even when out of their indications. Our study suggested that several aspects should be taken into consideration regarding the off‐label use of targeted drugs. First, the efficacy of the matched drug may not been validated in breast cancer, although it may have been proved to be effective in other specific tumor types. Given the complex signaling pathway network in tumor cells, these drugs may not be an ideal inhibitor for the target detected across different conditions. Second, the safety profile of off‐label drug is uncertain in breast cancer, and server adverse events may occur especially when a combination treatment strategy was adopted. Third, mutations of the same gene may play distinct roles across different tumor types or tumor histological types. Similarly, different alteration types or sites of the same gene may also have multifarious sensitivity to the same targeted drug. In addition, accompanying mutations in other genes, changes on epigenetics, transcriptomics, and proteomics level could also be unknown factors influencing the efficacy of the treatment. Taken together, physicians should be cautious about the off‐label use of targeted drugs.

Limitations of this study include the retrospective nature of the analysis, the small sample size, the unbalanced baseline characteristics of patients, and other unknown confounding factors, which may exert an effect on the survival. Heavily pretreated patients and the coexistence of driver mutations involved in other pathways may also have weakened the efficacy of everolimus treatment, which were not taken into consideration. Future studies in a larger cohort is warranted for further exploration of the efficacy of genotype‐directed treatment.

In conclusion, the molecularly matched off‐label use of everolimus might not provide sufficient benefit for refractory breast cancer patients harboring *PI3K/ATK/mTOR* pathway alterations.

## CONFLICT OF INTEREST

Yuzi Zhang, Zhengyi Zhao, Guoqiang Wang, Jing Zhao and Shangli Cai are employees of 3D Medicines Inc. Other authors declare that they have no conflict of interest.

## Data Availability

The data that support the findings of this study are available on request from the corresponding author. The data are not publicly available due to privacy or ethical restrictions.
