# Supplementary material for: Everolimus‐containing therapy vs conventional therapy in the treatment of refractory breast cancer patients with PI3K/AKT/mTOR mutations: A retrospective study
Source: Cancer Med. 2019 Aug 6;8(12):5544–53. doi: 10.1002/cam4.2460 (PMC6745827; doi:10.1002/cam4.2460)
Supplement: Supplementary file 1 [file CAM4-8-5544-s001.docx]

**Supplement**

**Table S1. PI3K/AKT/mTOR pathway genes list**

| AKT1 | AKT2 | AKT3 | MTOR | RICTOR | RPTOR | TSC1 | TSC2 |
| --- | --- | --- | --- | --- | --- | --- | --- |
| STK11 | PIK3CA | PIK3CG | PIK3C3 | PIK3R2 | PIK3C2B | PIK3CB | PIK3CD |
| PTEN | PIK3R1 | VHL |  |  |  |  |  |

**Table S2. Inclusion and exclusion criteria**

| **Inclusion Criteria** | |  | |
| --- | --- | --- | --- |
| *I* | Refractory breast cancer patients progressed after systemic chemotherapy for metastatic disease. | | |
| *II* | Received molecular screening using NGS with the aim of guiding treatment after confirmed disease progression between July 7, 2015 and October 30, 2017. | | |
| *III* | At least one gene mutation which could result in *PI3K/AKT/mTOR* pathway activation. | | |
| *IV* | Received everolimus off-label treatment or conventional treatment after NGS test. | | |
| *V* | Patients with HER2 positive breast cancer should progressed after anti-HER2 therapy. | | |
| **Exclusion Criteria** | | |  |
| *I* | Standard everolimus treatment. | | |
| *II* | Patients with HER2 positive breast cancer received treatment which did not combined with anti-HER2 therapy or were treated with anti-HER2 monotherapy. | | |

**Table S3. Treatment received by each patients in the everolimus group.**

| **Subject ID** | **Everolimus Therapy Received** |
| --- | --- |
| 001 | Everolimus + Trastuzumab + Vinorelbine |
| 006 | Everolimus + Albumin-bound paclitaxel + Oxaliplatin |
| 007 | Everolimus + Trastuzumab |
| 008 | Everolimus + Vinorelbine + Trastuzumab |
| 009 | Everolimus + Vinorelbine +Trastuzumab |
| 010 | Everolimus + Vinorelbine + Trastuzumab |
| 011 | Everolimus + Vinorelbine + Trastuzumab |
| 012 | Everolimus + Letrozole |
| 013 | Everolimus + Vinorelbine + Capecitabine |
| 014 | Everolimus + Vinorelbine + Trastuzumab |
| 021 | Everolimus + Trastuzumab |
| 022 | Everolimus + Gemcitabine |
| 023 | Everolimus + Lapatinib + Gemcitabine |
| 024 | Everolimus |
| 025 | Everolimus + Paclitaxel + Trastuzumab |
| 027 | Everolimus + Docetaxel +Trastuzumab |
| 028 | Everolimus + Capecitabine |
| 029 | Everolimus + Carboplatin |
| 032 | Everolimus + Toremifene |

**Table S4. Mutations in *PI3K/AKT/mTOR* pathway.**

| **ID** | **Molecular subtype** | **Mutation genes** | **cDNA change** | **Amino acid change** |
| --- | --- | --- | --- | --- |
| 001 | HR-/HER2+ | PIK3CA | c.A3140G | p.H1047R |
| 002 | HR-/HER2+ | PIK3CA  TSC1 | c.A3130T  c.C1697T | p.N1044Y  p.P566L |
| 003 | HR+/HER2- | PIK3CA  PIK3CA  PIK3CA | c.G1633A  c.G2176A  c.G2702T | p.E545K  p.E726K  p.C901F |
| 004 | HR-/HER2+ | PIK3CA | c.A1034C | p.N345T |
| 005 | TNBC | PIK3CA | c.A3140G | p.H1047R |
| 006 | TNBC | PIK3CA | c.A3140G | p.H1047R |
| 007 | HR+/HER2+ | PIK3CA  PIK3CB  MTOR | c.A3140G  c.1438T  c.G175A | p.H1047R  p.Q480*  p.E59K |
| 008 | HR-/HER2+ | VHL |  | Loss |
| 009 | HR+/HER2+ | PIK3CA  PIK3CA | c.A3140G  c.G1633C | p.H1047R  p.E545Q |
| 010 | HR+/HER2+ | PIK3CA  MTOR | c.A3140G  c.C3703T | p.H1047R  p.R1235W |
| 011 | HR-/HER2+ | PIK3CA | c.A3140G | p.H1047R |
| 012 | HR+/HER2- | PIK3CA | c.A3140G | p.H1047R |
| 013 | TNBC | STK11 |  | Loss |
| 014 | HR-/HER2+ | PIK3CA  PTEN  AKT3 | c.A3140G  c.G70C  c.615T＞A | p.H1047R  p.D24H  p.H205Q |
| 015 | HR+/HER2- | PIK3CA | c.A3140G | p.H1047R |
| 016 | HR+/HER2- | PIK3R1 | c.1354_1355delinsG | p.Y452Vfs*28 |
| 017 | TNBC | PIK3CA | c.G1624A | p.E542K |
| 018 | HR+/HER2- | PIK3CA  PIK3CA | c.A3140G  c.G1635T | p.H1047R  p.E545D |
| 019 | HR+/HER2- | PIK3CA | c.G1633A | p.E545K |
| 020 | HR+/HER2- | PIK3CA | c.G1624A | p.E542K |
| 021 | HR-/HER2+ | PIK3CA | c.A3140G | p.H1047R |
| 022 | TNBC | PIK3CA  PIK3C2B  PTEN | c.G1624A  c.A512G | p.E542K  Amplification  p.Q171R |
| 023 | HR-/HER2+ | PIK3CA  AKT1 | c.G1633A | p.E545K  Amplification |
| 024 | TNBC | PIK3CA | c.T1258C | p.C420R |
| 025 | HR+/HER2+ | PIK3R1 | c.373_374insCTGA | p.I127* |
| 026 | HR+/HER2+ | PIK3CA  AKT1  TSC2 | c.G1633A  c.C4818A | p.E545K  Loss  p.F1606L |
| 027 | HR+/HER2+ | PIK3CA | c.A3140G | p.H1047R |
| 028 | HR+/HER2- | PIK3CA | c.G1633A | p.E545K |
| 029 | TNBC | PIK3CA | c.G2702C | p.C901S |
| 030 | HR+/HER2- | PIK3CA | c.A331G | p.K111E |
| 031 | HR+/HER2- | PIK3CA | c.A3140G | p.H1047R |
| 032 | HR+/HER2- | PIK3CA | c.A3140G | p.H1047R |
